# A complication of the transradial approach: thyrocervical trunk pseudoaneurysm with hemothorax

**DOI:** 10.1259/bjrcr.20220136

**Published:** 2023-05-03

**Authors:** Kareem El Naamani, Rawad Abbas, Georgios S Sioutas, Abdelaziz Amllay, Stavropoula Tjoumakaris, Michael R Gooch, Robert H Rosenwasser, Pascal Jabbour

**Affiliations:** 1 Department of Neurological Surgery, Thomas Jefferson University Hospital, Philadelphia, Pennsylvania, USA

## Abstract

The transradial approach has gained popularity in the neuroendovascular field after several studies proved its low rate of hemorrhagic and vascular-related complications in both diagnostic and therapeutic procedures.

This is a case of a patient who presented for flow diversion treatment of an incidental left carotid ophthalmic aneurysm. The procedure was uneventful. Post-operatively, the patient’s neurological exam and vital signs were normal, however the patient complained of abdominal and chest pain that worsened when lying down and improved when sitting up. Radiologic diagnosis confirmed the presence of a thyrocervical trunk pseudoaneurysm which was completely obliterated with Onyx 18 embolization. Thyrocervical trunk pseudoaneurysm formation is a rare complication of the transradial approach. Shedding the light on these entities is essential as symptoms vary in severity and presentation and necessitate swift diagnosis and treatment.

## Introduction

The transradial approach has gained popularity in the neuroendovascular field after several studies proved its low rate of hemorrhagic and vascular-related complications in both diagnostic and therapeutic procedures.^1,2^ However, the transradial approach is not free of complications.^3,4^ Complications vary in severity, presentation, location and incidence and include radial artery occlusion, spasm, perforation, pseudoaneurysm formation and arteriovenous fistula formation at the access site. However, other non-access site complications can occur during procedures. One of which is a thyrocervical trunk pseudoaneurysm formation which has only been reported twice in the cardiac literature. In this short report, we report the first neuroendovascular case of thyrocervical trunk pseudoaneurysm formation following a transradial approach aneurysm flow diversion treatment.

## Report

### Clinical presentation

This is a case of a patient who presented for flow diversion treatment of a left carotid ophthalmic aneurysm that was incidentally discovered on magnetic resonance angiography (MRA).

### Procedure details

**Figure 1.**
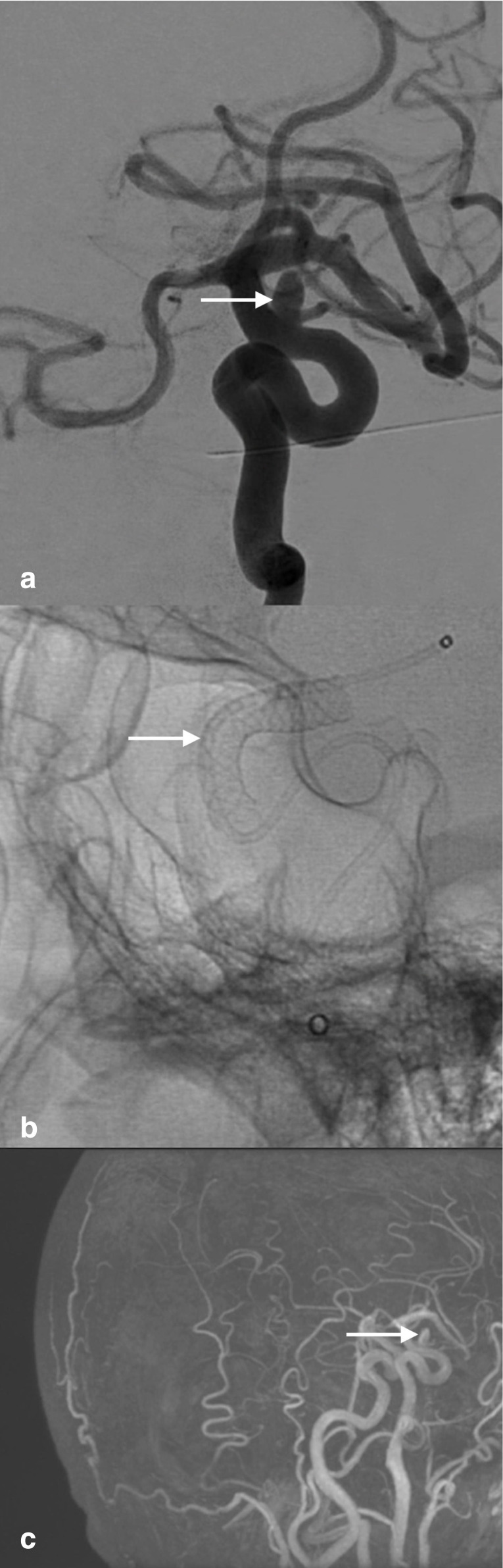
(a) Diagnostic angiography (lateral view) showing a left carotid ophthalmic artery aneurysm; (**b** fluoroscopy (lateral view) showing the pipeline shield implantation. (**c**) 3-dimensional magnetic resonance angiography showing a left carotid ophthalmic artery aneurysm.

The right radial artery was catheterized with a 088 Ballast (Balt, Montmorency, France) through which a 0.038 Terumo guidewire and a Simmons two catheter were introduced. Both catheters were advanced under fluoroscopic guidance with the Glidewire inside, all the way to the arch. At no time, the glidewire was noticed to buckle or go inadvertently into any of the arterial branches. The Simmons 2 was shaped in the arch, and the left internal carotid artery was selectively catheterized with the Simmons 2. Then, the 088 Ballast was advanced over the Simmons 2 into the left internal carotid artery. The left middle cerebral artery (M1) was super selectively catheterized with a Phenom 27 (Medtronic, Dublin, Ireland) and a Synchro 2. A 4.25 × 14 mm Pipeline Embolization Device (PED) with Shield technology (Medtronic, Dublin, Ireland) was deployed across the aneurysm neck. Finally, a left carotid artery injection showed adequate placement of the PED. (Figure 1a-c)

### Post-procedure

The procedure was uneventful, and the patient was on a dual antiplatelet regimen of Plavix 75 mg and Aspirin 81 mg. 24 h post-operatively, the patient’s neurological exam and vital signs were normal, however the patient complained of abdominal and chest pain that worsened when lying down and improved when sitting up. Cardiac work-up was normal and the patient denied any difficulty breathing.

### Diagnosis and imaging

**Figure 2.**
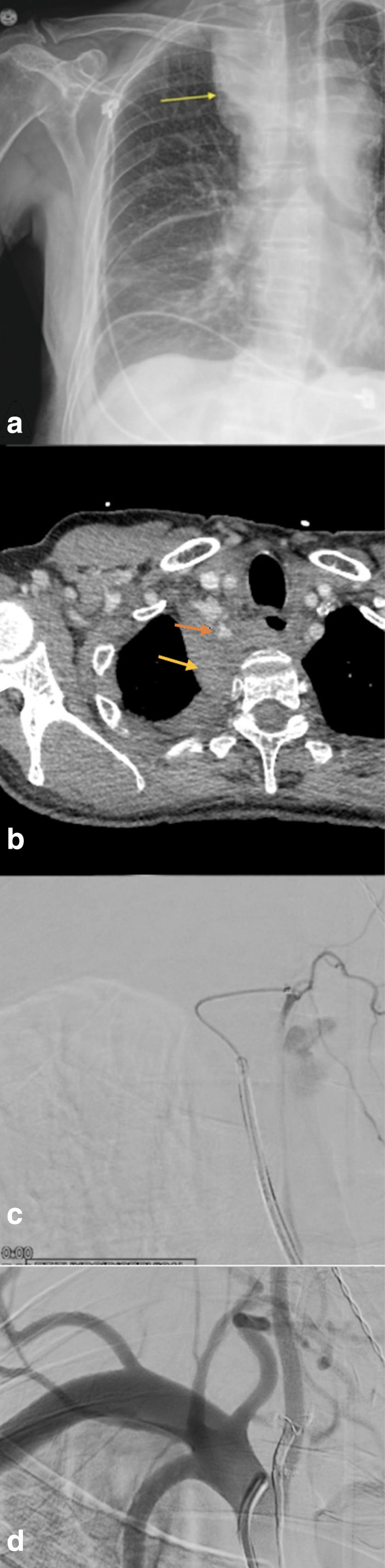
(a) Anteroposterior chest X-ray showing right upper peri mediastinal opacity (Yellow Arrow). (**b)** Contrast-enhanced chest CT scan demonstrating a right hemomediastinum and right hemothorax (Red Arrows). (**c)** A superselective injection showing the thyrocervical trunk pseudoaneurysm. (**d)** Fluoroscopy showing complete obliteration of the pseudoaneurysm after Onyx embolization.

On chest X-ray, a 7 cm right upper peri mediastinal opacity without any pneumothorax or pleural effusion was noted. (Figure 2a) Further investigation with an abdominal and chest contrast-enhanced CT scan showed right hemomediastinum, right hemothorax and right subclavian extravasation suggestive of a pseudoaneurysm (Figure 2b). At this point, the patient was taken to the angio suite for a digital subtraction angiography (DSA) to assess and treat any arterial injury. A transfemoral angiography was performed and confirmed a right thyrocervical trunk pseudoaneurysm which was embolized with 0.3 cc of Onyx 18 (Onyx Liquid Embolic System, Micro Therapeutics, Inc., Irvine, CA) (Figure 2c). A selective right subclavian artery control angiogram showed complete occlusion of the pseudoaneurysm with no pooling of contrast (Figure 2d).

### Hospital course and discharge

During the post-operative period, the patient’s physical exam was normal otherwise and vitals were stable. The next day the patient was discharged home on the aforementioned dual antiplatelet regimen.

## Discussion

As minimally invasive endovascular procedures are becoming more popular worldwide, these procedures pose both systemic and treatment-located local risks.^5^ Pseudoaneurysm formation in neuroendovascular procedures is rare and multifactorial most often due to dissections caused by the wire or the guide catheter.^5–7^ Because intravascular wires pass from the radial artery to intracranial vessels, dissections can occur at any location, and pseudoaneurysms may form anywhere along the radial route. In this case report, injury to the thyrocervical trunk by the catheter resulted in the formation of a pseudoaneurysm. The thyrocervical trunk is one of three branches of the first part of the subclavian artery. Arising lateral to the vertebral artery, the thyrocervical trunk gives rise to the inferior thyroid artery, suprascapular artery, ascending cervical artery and transverse cervical artery. Patients with pseudoaneurysms can be symptomatic according to the anatomical location of the pseudoaneurysm and diagnosis is usually based on doppler ultrasonography for superficial lesions and CT angiography for deep lesions.^8^ In most cases, pseudoaneurysms spontaneously thrombose, however untreated active cases may lead to infection or rupture.^5^ Management of mild cases is escalated from compression to ultrasound-guided compression of the pseudoaneurysm neck and ultrasound-guided thrombin injection in superficial cases while surgical intervention or embolization are performed in deep cases.^7^

To our knowledge, thyrocervical trunk pseudoaneurysm formation has not been reported in the neuroendovascular literature, and only two case reports in the cardiac literature have discussed this complication. In 2012, Villaneuva-Benito et al reported a case of an iatrogenic subclavian artery pseudoaneurysm after a patient underwent a transradial coronary stenting for a non-ST-segment elevation acute coronary syndrome.^9^ One day after the procedure, the patient complained of excruciating pain in the right hemithorax radiating to the right forearm. A computed tomogram was performed and confirmed the diagnosis. The pseudoaneurysm was treated with a self-expanding stent and the patient was discharged home uneventfully.^9^

In 2017, Alpay et al reported a case of thyrocervical trunk pseudoaneurysm formation after a patient underwent a diagnostic transradial coronary angiography.^10^ Immediately after the removal of the radial sheath, a swelling in the supraclavicular area was noticed and a duplex ultrasonography and computed tomography angiography (CTA) confirmed the diagnosis. The pseudoaneurysm thrombosed after several cycles of ultrasound-guided aneurysm neck compressions and the patient was discharged home uneventfully.^10^ Presenting symptoms may vary depending on the location and rupture status of the pseudoaneurysm. In our case report, like Villaneuva-Benito et al, the deep location and active nature of the pseudoaneurysm resulted in accumulation of blood in the hemithorax and mediastinal region causing chest pain and requiring a more aggressive treatment option compared to Alpay et al.’s pseudoaneurysm superficial location and asymptomatic presentation in which a less invasive treatment modality sufficed.^9,10^ Also, because of the rarity of such a complication, no guidelines are present to direct treatment options which was evident in the three different approaches of treatment.

In our case report, when accessing a left intracranial aneurysm through the right radial artery, catheters and glide wires pass through the right subclavian artery and the Simmons 2 catheter is shaped in the aortic arch before catheterizing the left common carotid artery. We believe that the glide wire went into the right thyrocervical trunk while passing through the right subclavian artery and caused the traumatic pseudoaneurysm. Slow and careful navigation through the subclavian artery is essential to avoid unwanted catheterization of the thyrocervical trunk.

## Learning points

- In our case report, the formation of the pseudoaneurysm was due to the dissection of the thyrocervical trunk by the glidewire.
- The importance of this case report is not only to acknowledge that thyrocervical trunk pseudoaneurysms, though extremely rare, can be a complication of the transradial approach but also, to shed the light on the probable presenting symptoms, diagnostic modalities and different treatment options used to provide quick and effective care.
- Also, it is imperative to keep a low threshold for suspecting such entities after transradial procedures especially for neurointervantionalists starting a radial first approach.

## Conclusion

Thyrocervical trunk pseudoaneurysm formation is a rare complication of the transradial approach. Shedding the light on these entities is essential as symptoms vary in severity and presentation and necessitate swift diagnosis and treatment.
